# Modelling ligand depletion for simultaneous affinity and binding site quantification on cells and tissue

**DOI:** 10.1038/s41598-023-37015-1

**Published:** 2023-06-20

**Authors:** Judith Weber, Klara Djurberg, Sara Lundsten Salomonsson, Maria Kamprath, Aileen Hoehne, Hadis Westin, Fernanda Vergara, Sina Bondza

**Affiliations:** 1 3B Pharmaceuticals GmbH, Berlin, Germany; 2 Ridgeview Instruments AB, Uppsala, Sweden; 3 Department of Immunology, Genetics and Pathology, Uppsala University, Uppsala, Sweden

**Keywords:** Biophysical methods, Assay systems, Drug discovery, Kinetics

## Abstract

The quantification of the number of targets in biological systems is an important parameter to assess the suitability of surface markers as targets for drugs, drug delivery and medical imaging. Likewise, quantifying the interaction with the target in terms of affinity and binding kinetics is essential during drug development. Commonly used approaches to quantify membrane antigens on live cells are based on manual saturation techniques that are labour-intensive, require careful calibration of the generated signal and do not quantify the binding rates. Here, we present how measuring interactions in real-time on live cells and tissue under ligand depletion conditions can be used to simultaneously quantify the kinetic binding parameters as well as the number of available binding sites in a biological system. Suitable assay design was explored with simulated data and feasibility of the method verified with experimental data for exemplary low molecular weight peptide and antibody radiotracers as well as fluorescent antibodies. In addition to revealing the number of accessible target sites and improving the accuracy of binding kinetics and affinities, the presented method does not require knowledge about the absolute signal generated per ligand molecule. This enables a simplified workflow for use with both radioligands and fluorescent binders.

## Introduction

Many cell surface proteins interact with specific extracellular ligands and are involved in a broad range of cellular functions. To understand the biological and pathological functions driven by a cell surface molecule, it is important to understand its expression on a cellular level. Most of these functions are linked to the surface molecule interacting with extracellular ligands, often triggering cellular signalling. The kinetics and affinity of these interactions constitute important knowledge for understanding and targeting signalling pathways. Quantifying the expression of surface molecules as well as their interactions with biological active compounds are thus crucial for investigating the suitability of surface molecules as drug targets or (imaging) biomarkers^1–3^. As the number and accessibility of the targeted surface structure can change rapidly due to several factors such as shedding, internalization and interactions with intrinsic binding partners, the examination of the available number of binding sites in the test environment is of great value^4^. Likewise, the binding rates with which a ligand interacts with the surface target also impact the biological response and are influenced by the cellular environment^2,5,6^. Therefore, a method quantifying binding kinetics and the number of accessible target sites in the same assay improves biological accuracy and relevance, especially when trying to relate binding properties to functional effects.

However, quantifying the number of targets on the cell surface of living cells while simultaneously monitoring ligand-target interactions in real-time is challenging. Standard methods applied to investigate the number of expressed molecules per cell include manual saturation measurements with radiolabelled compounds, immunoblotting and flow cytometry^7^. Most of these methods are either indirect approaches, prone to errors as the required receptor saturation is not always reached^8^ or are labour-intensive and complex^9^. Moreover, many of these methods determine the number of receptors on separated cells and thus do not provide an insight into the binding site availability in a cellular network^10^. Previously, a method called the kinetic extrapolation method (KEX) has been reported that uses real-time measurements of ligand-receptor interactions on live cells as an addition to manual measurements to remove the need for reaching receptor saturation while also providing the rate constants of the binder^11^. Essentially, KEX takes the data from a real-time interaction measurement and extrapolates the level of target occupancy to determine what the signal at receptor saturation would be. However, KEX requires the signal contribution per ligand molecule to be known and is thus largely limited to radioligands. In more recent years advanced and often complex set-ups have combined real-time binding measurements with technologies that allow visualization of cell surface molecules to obtain an estimation of receptor density, although typically using fixated cells^12,13^.

An alternative method to quantify the number of cell surface targets on live cells that does not rely on knowing the signal/concentration ratio is utilizing ligand depletion during real-time ligand-target interaction measurements. Ligand depletion is the noticeable reduction of free ligand concentration as a direct result of receptor binding. Thus, if ligand depletion is present, the assumption of near constant ligand concentration during the binding assay is no longer valid^8^. Modelling this reduction of free ligand concentration allows to calculate the number of available binding sites on the sample surface in a fast and simple approach not limited to radioligands. Many novel therapeutics exhibit extremely strong binding to their targets with affinities frequently in the sub-nanomolar region. Especially these strong binders are prone to ligand depletion effects, as cell-based saturation binding assays are commonly performed as end-point measurements in closed systems with ligand concentrations from tenfold lower to tenfold higher than the affinity value, which makes ligand depletion hard to avoid and thus should be accounted for when analysing such data^8,14^. Ligand depletion is further enhanced when using model systems with high target expression which are typically applied during drug development^15^. Conversely, ligand depletion can be purposefully introduced by increasing the number of cells or lowering the ligand concentration. Apart from being able to deduce the number of targets accessible for binding, modelling ligand depletion is also needed for accurate determination of binding affinities and kinetics of strong binders especially for high expressing model systems^14^.

In the current study, we addressed the need for a fast, simple and accurate method to simultaneously characterise ligand-target interactions and quantify the number of targets in real-time on living cells and tissues. We first investigated the influence of ligand depletion on the relative error of on- and off-rate estimates and thus affinity quantification when constant ligand concentrations are assumed. Secondly, we explored suitable experimental parameters, such as number of ligand concentrations being measured and the degree of depletion needed for reliable receptor quantification through depletion-based kinetic modelling. Suitable assay design and feasibility of the method were verified on both living cells and tissue for low molecular weight, peptidic and antibody radiotracers as well as for fluorescent antibodies. In conclusion, we demonstrate that by measuring binding interactions in real-time under ligand-depleting conditions and applying depletion-based modelling both the kinetics of the binder as well as the number of available binding sites can be accurately quantified.

## Results

### Influence of ligand depletion on accuracy of kinetic parameter extraction

**Figure 1:**
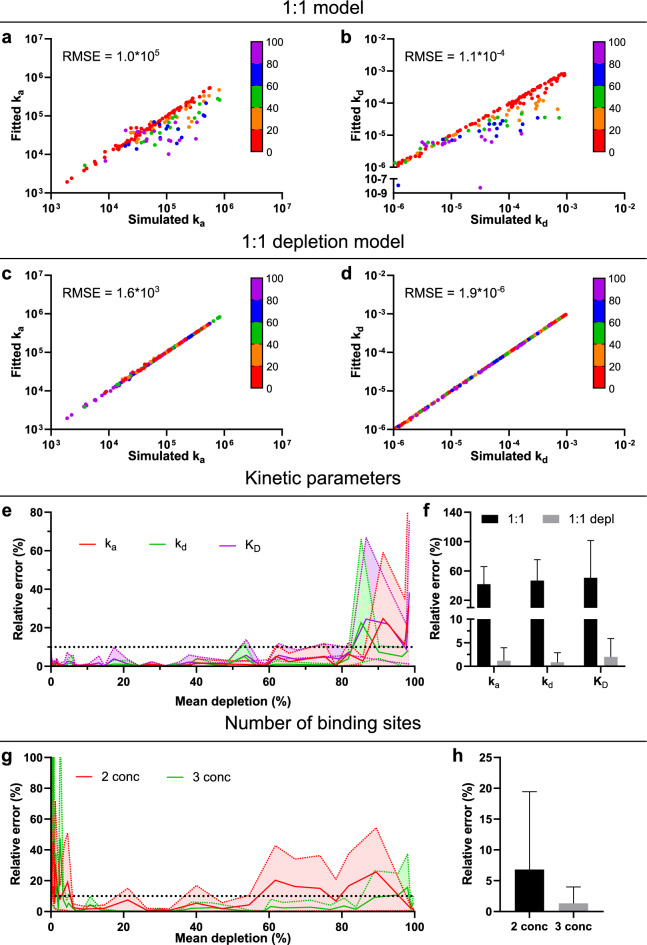
Analysis of simulated data set containing varying degrees of ligand depletion. Comparison of 1:1 and 1:1 depletion models (**a–d**) and investigation of the relative error for kinetic parameters (**e,f**) and number of binding sites (**g,h**). Fitted versus simulated k_a_ (M^−1^ s^−1^, n = 153) (**a**), k_d_ (s^−1^, n = 153) (**b**) using the 1:1 model. Fitted versus simulated k_a_ (M^-1^ s^-1^, n = 234) (**c**), k_d_ (s^−1^, n = 231) (**d**) using the 1:1 depletion model. The coloring of data points in (**a–d**) depicts the mean depletion (%) of each simulation. Relative error plotted against mean depletion for k_a_, k_d_ and K_D_ (n = 5 for each data point) (**e**). Shadowed area depicts the s.d. The relative error for k_a_, k_d_ and K_D_ when the mean depletion is between 10 and 80% (mean ± s.d., n ≥ 63) using the 1:1 or 1:1 depletion model (**f**). Relative error plotted against mean depletion for the number of binding sites when having 2 or 3 concentrations in the association phase (n = 5 for each data point) (**g**). Shadowed area depicts the s.d. The mean relative error for number of binding sites when the mean depletion is between 10 and 80% (mean ± s.d., n ≥ 55) (**f**). s.d. = standard deviation.

As shown previously, real-time binding data needs to contain two association phases with sufficient curvature recorded at different ligand concentrations and a dissociation phase for reliably extracting kinetic information with the 1:1 model^16^. For the more complex depletion model, we hypothesized three ligand concentrations to be necessary for accurate parameter extraction. Therefore, real-time binding curves with three increasing ligand concentrations and a dissociation phase were simulated with differing degrees of ligand depletion (for details see materials and methods). The dataset consisting of 244 binding traces was then fitted with both the standard 1:1 model assuming constant ligand concentration for each association phase and the 1:1 model with correction for ligand depletion. The relative error of the association rate constant k_a_ and dissociation rate constant k_d_ were plotted against the average depletion of the simulated binding curves (Fig. 1a–d). The 1:1 model predicts slower k_a_ (Fig. 1a) in the presence of ligand depletion and this underestimation of k_a_ becomes noticeable at a mean ligand depletion (D_mean_) of 20%. The k_d_ is underestimated by the 1:1 model as well and the effect becomes even more noticeable with increasing D_mean_ (Fig. 1b). As seen in the root mean square error (RMSE) values, both rate constants are more accurately estimated when applying the 1:1 depletion corrected model (Fig. 1c,d). Furthermore, a D_mean_ above 80% worsens the accuracy with which the rate constants and thus affinity can be determined with the depletion-corrected model (Fig. 1e), implying that high levels of ligand depletion in all association phases is suboptimal for parameter determination. If the D_mean_ is limited to 10–80%, the mean relative error of k_a_, k_d_ and K_D_ (Fig. 1f) are 41, 47 and 51% respectively using the 1:1 model. In contrast, the errors when using the depletion-corrected model are 1.2, 0.8 and 2.0% for the same parameters.

### Using ligand depletion to determine number of binding sites

When using the 1:1 depletion corrected model, ligand depletion is assumed to occur through target binding. As this ligand depletion is visible in the shape of the binding trace it, and thereby also the number of bound targets, can be estimated. Combining this with the knowledge of the signal at saturation (B_max_) the total number of binding sites (nB) can be calculated. To understand whether three concentrations are indeed necessary for using the depletion model to reliably extract nB, we complemented our previous data set with an additional data set simulated with two ligand concentrations. A D_mean_ above approximately 5% is required to estimate nB with a relative error of 10% or less when using both two and three ligand concentrations (Fig. 1g). Furthermore, a high average depletion also negatively impacts the accuracy of nB, similar to what was seen for the kinetic parameters. This was especially pronounced when using only two ligand concentrations, where a D_mean_ above approximately 55% results in a relative error of 10% or higher. Limiting the D_mean_ to 10–80%, the mean relative error of nB (Fig. 1h) is 6.8% using two concentrations and 1.3% using three concentrations for simulated data. Based on these findings and the presumption that error margins are higher for experimental data, three concentrations during the association and a dissociation phase were deemed a suitable experimental set-up moving forward.

### Quantifying binding kinetics and number of cell surface binding sites for radioligands

**Figure 2:**
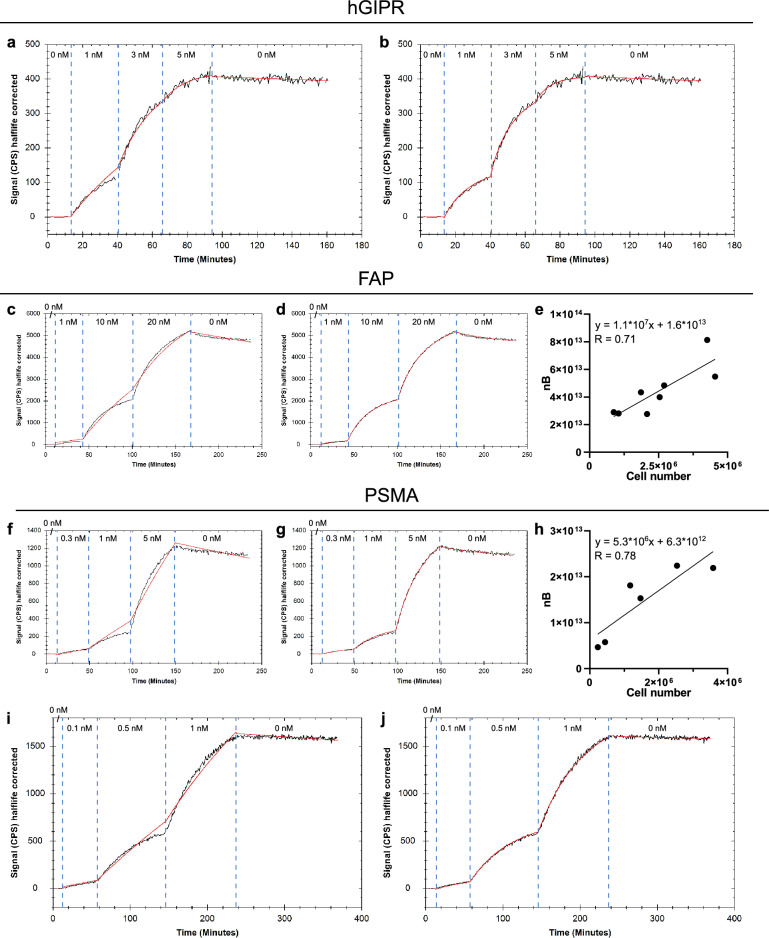
Representative binding traces for radioligands. Peptide tracer binding to human GIPR-expressing CHO cells (**a,b**), peptide tracer binding to FAP-expressing HEK cells (**c,d**), low molecular weight tracer binding to PSMA-expressing PC3-PIP cells (**f,g**) and anti-PSMA antibody binding to PSMA-expressing PC3-PIP cells (**i,j**). Binding traces are displayed in black and fitted lines in red: fitted with the 1:1 model (**a,c,f,i**) and with the 1:1 depletion corrected model (**b,d,g,j**), respectively. Cell number used per replicate plotted against number of binding sites (nB) estimated by the depletion model for the corresponding model system (**e,h**).

The simultaneous assessment of ligand kinetics and quantification of the receptor density was also performed in an experimental setting using a collection of radiolabelled ligands. Real-time binding traces (Fig. 2a–e) were first recorded for peptidic radiotracers targeting GIPR (GIPR-Tracer), and FAP (FAP-Tracer). The resulting interaction traces from three increasing ligand concentrations and a dissociation phase were fitted to a standard 1:1 model (Fig. 2a,c) as well as the depletion corrected 1:1 model (Fig. 2b,d). The 1:1 model produced a fit with less curvature than was present in the first association phase of the measured data for both tracers. Moreover, this effect was also evident in the two subsequent association phases for the FAP-Tracer (Fig. 2c). This is a typical sign of ligand depletion: as the free ligand concentration reduces during the association phase, the binding slows down resulting in curvature that is not predicted by the 1:1 model. When applying the 1:1 depletion-corrected model this reduction in ligand concentration was taken into account and thus the curvature of the measured data could be accurately represented.

The rate constants (k_a_ and k_d_) were extracted from the depletion model for all tested tracers (Table 1). Furthermore, the number of total receptors was extracted and normalized to the total cell number present in each experiment. For the GIPR-Tracer, the affinity was determined to be 17 pM (Table 1). While the variation in k_a_ was low between replicates, the very slow k_d_ shows, as expected, a higher variability. This is also reflected in the affinity value. The number of receptors per cell was 1.1 × 10^6^, which is higher compared to the number determined by the KEX method for which an average receptor count of 6.4 × 10^5^ per cell was calculated. Extraction of the kinetic parameters using the depletion model resulted in an affinity value of 2.3 nM for the FAP-Tracer. The depletion model estimated the number of molecules/cell to 1.8 × 10^7^, compared to 1.3 × 10^7^ molecules/cell calculated through the KEX method.

**Table 1:** Summary of kinetic parameters and receptor quantification.

Model system	n	Depletion model				KEX
		k_a_ (M^−1^ s^−1^)	k_d_ (s^−1^)	K_D_ (M)	No. receptor/cell	No. receptor/cell
GIPR^A^	5	5.35E + 05 (16%)	8.85E − 06 (51%)	1.73E − 11 (53%)	1.14E + 06 (28%)	6.40E + 05 (12%)
FAP^A^	5	1.74E + 04 (16%)	3.85E − 05 (41%)	2.25E − 09 (46%)	1.78E + 07 (16%)	1.33E + 07 (17%)
FAP^B^	8	1.90E + 04 (19%)	4.41E − 05 (26%)	2.34E − 09 (24%)	2.02E + 07 (36%)	1.61E + 07 (26%)
PSMA^B^	6	8.15E + 04 (63%)	4.82E − 05 (48%)	6.59E − 10 (43%)	1.23E + 07 (40%)	7.99E + 06 (38%)
PSMA-AB^A^	3	5.04E + 04 (13%)	5.79E − 06 (23%)	1.17E − 10 (32%)	3.84E + 06 (13%)	3.66E + 06 (30%)
PERTUZUMAB^A^	5	1.30E + 05 (7%)	3.94E − 06 (52%)	2.98E − 11 (46%)	1.88E + 06 (23%)	n.a
TRASTUZUMAB^A^	5	1.43E + 05 (10%)	5.27E − 06 (56%)	3.55E − 11 (43%)	2.06E + 06 (18%)	n.a

Mean values (with CV) of k_a_, k_d_, K_D_ and number of receptors per cell for each model system using depletion model and/or KEX.

*CV* coefficient of variations, *n.a.* not available.

^A^Seeding a constant number of cells for each experiment.

^B^Varying cell number between experiments.

In a second set of experiments using the FAP model system, the cell number used per experiment was varied. Quantifying the binding with the depletion model resulted in the same affinity value of 2.3 nM, however the calculations of target sites/cell showed greater variability compared to the previous experiments that were performed with confluent cells at the time of the assay (Table 1). Nevertheless, as expected, the number of total binding sites predicted by the depletion model correlated with the total number of cells present in the experiment (Fig. 2e).

The method was further assessed using a PSMA-expressing prostate cancer cell line (PC3-PIP) as a model system. A Glu-ureido–based PSMA-Tracer was evaluated using varying cell numbers (Fig. 2f–h), as well as an antibody (PSMA-Ab) using constant cell numbers (Fig. 2i,j). The 1:1 depletion model (Fig. 2g,j) could fit the recorded binding traces noticeably better compared to the standard 1:1 model (Fig. 2f,i), similarly to the previous model systems. As expected, the presence of more cells during the experiment resulted in higher number of total binding sites calculated through the depletion model (Fig. 2h).

Using the depletion-corrected 1:1 model, an affinity of 0.7 and 0.1 nM was calculated for the PSMA-Tracer and PSMA-Ab, respectively (Table 1). For the PSMA-Tracer, the receptor cell count was estimated to 1.2 × 10^7^ using the depletion model and 8.0 × 10^6^ for KEX. The binding capacity of the antibody was 3.8 × 10^6^ molecules/cell, which was in good agreement with the KEX estimate of 3.6 × 10^6^ molecules/cell.

### Modelling ligand depletion to determine the number of binding sites on tissue

**Figure 3:**
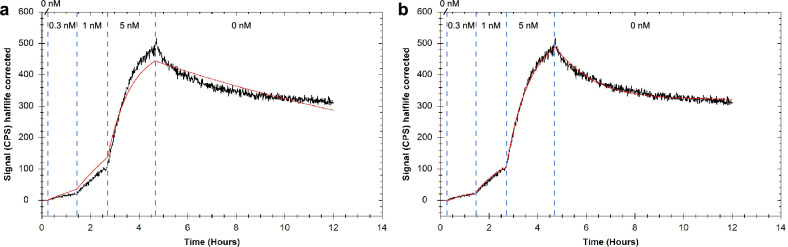
Representative binding trace for the low molecular weight PSMA-tracer to PSMA-expressing PC3-PIP xenograft tissue. Binding traces are displayed in black and fitted lines in red: fitted with the 1:1 model (**a**) and with the 1:1 depletion corrected model (**b**), respectively.

For the evaluation of biological active compounds, tissues are important model systems. Thus, being able to determine binding parameters in real-time simultaneously with the number of accessible binding sites on the tissue samples in a simple and fast way would be of great benefit. Hence, we tested our method on PC3-PIP xenograft tissue using the Glu-ureido–based PSMA-Tracer (Fig. 3), which was used in previous experiments on PC3-PIP cells. Applying the depletion corrected 1:1 model, the fit mirrored the experimental traces allowing a precise characterization of the compound on tissue (Table 2). Further, through fitting, an estimate of the accessible binding sites on the tissue could be obtained, which did range between 3.38 × 10^12^ and 5.02 × 10^13^ receptors per tissue sample.

**Table 2:** Summary of kinetic parameters (k_a_, k_d_, K_D_) and receptor quantification (nB) of the ^111^In-PSMA-Tracer on PC3-PIP xenograft tissue samples.

Tissue ID	Depletion model			
	k_a_ (M^−1^ s^−1^)	k_d_ (s^−1^)	K_D_ (M)	No. receptor/sample
10	1.84E + 05	6.72E − 05	3.65E − 10	3.38E + 12
01	7.42E + 03	7.98E − 05	1.07E − 08	5.02E + 13
01	3.38E + 04	5.74E − 05	1.70E − 09	8.84E + 12
MEAN (CV)	7.51E + 04 (127%)	6.81E − 05 (17%)	4.26E − 09 (132%)	n.a.^A^

Tissue IDs refer to the xenograft sample that sections were obtained from, i.e. same tissue IDs reflect measurements of different sections from the same tissue sample. Results for each replicate as well as mean (CV) are presented.

*CV* coefficient of variations, *n.a.* not available.

^A^Mean number of receptors per sample are not calculated as tissue sections differ in size.

### Determining binding kinetics and capacity for therapeutic antibodies with fluorescent labels

**Figure 4:**
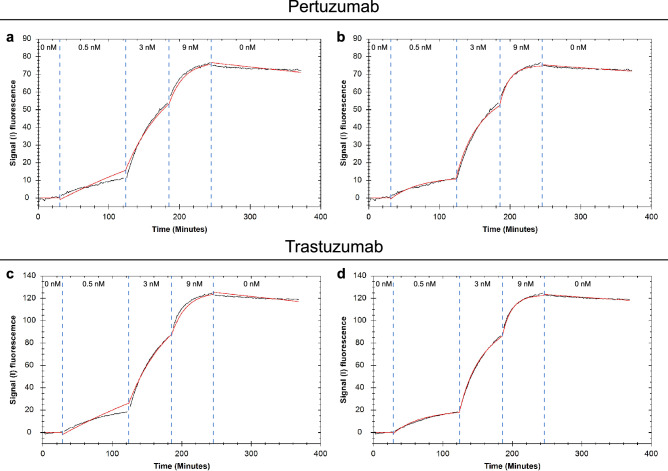
Representative binding traces for fluorescent antibodies. Atto488-Pertuzumab (**a,b**) and Atto488-Trastuzumab (**c,d**) binding to HER2-expressing SKOV3 cells. Binding traces are displayed in black and fitted lines in red: fitted with the 1:1 model (**a,c**) and with the 1:1 depletion corrected model (**b,d**), respectively.

To demonstrate that working with a radiolabel is not a necessity for estimating target number through depletion modelling, the two HER2 targeting antibodies Trastuzumab and Pertuzumab were fluorescently labelled and their binding to the HER2-overexpressing cancer cell line SKOV3 was recorded with LigandTracer Green. The first association phase of the resulting binding traces showed more curvature than expected for interactions behaving according to the 1:1 model (Fig. 4a,c), which, as mentioned above, is a typical sign of ligand depletion being most noticeable at the lowest concentration. The 1:1 depletion corrected model could accurately fit the data obtained for both antibodies (Fig. 4b,d) resulting in very strong apparent affinities of 36 pM for Trastuzumab and 30 pM for Pertuzumab. The calculated HER2 antibody binding capacity for SKOV3 cells were in good agreement between the antibodies with 2.1 × 10^6^ receptors/cells extracted from the Trastuzumab binding traces and 1.9 × 10^6^ receptors/cell estimated using the Pertuzumab binding data (Table 1). The reproducibility for parameter extraction using fluorescent data was comparable to data acquired with radioligands.

## Discussion

In this study, we present and validate the use of the 1:1 ligand depletion corrected binding model for simultaneously quantification of kinetic parameters and determination of the number of available binding sites on living cells and tissue.

For extracting kinetic binding parameters from real-time interaction curves with the standard 1:1 model, an assay set-up with two ligand concentrations with curvature and a dissociation phase is required^16^. Working with a simulated data set, we showed that three increasing ligand concentrations and a dissociation phase are sufficient to accurately determine the kinetic parameters as well as the number of binding sites nB with the 1:1 depletion corrected model, when the mean depletion (D_mean_) is between 10 and 80%. The observation that a minimal level of ligand depletion is needed to model the depletion effect and thus nB was expected. The decrease in accuracy of the depletion model with high levels of average ligand depletion was, in contrast, unexpected. Among the simulated data with an average depletion above 80%, we identified eight binding traces (including outliers) that had a relative error larger than 10% for calculating nB. The number of binding sites is closely related to the theoretical maximum signal B_max_ and for the aforementioned eight traces, the relative error for B_max_ was larger than 10%. The presence of high ligand depletion in all three association phases (i.e. resulting in a high D_mean_), led to the assumption that the target occupancy reached in those simulated experiments was probably quite low: Indeed, seven out of the eight traces reached a maximum target occupancy of below 25%, which affects the accuracy with which B_max_ can be determined. In summary, this means that a high D_mean_ in combination with low target occupancy reached in the experiment affects the accuracy of B_max_ and therefore also nB. Conversely, in our simulated data set we also found 26 binding traces with an average depletion above 80% and a relative error for nB below 10%. For all these traces the relative error for B_max_ was also below 10% and the maximum target occupancy reached in the simulated assays was 25% or higher. It should be noted, that, even for low levels of target occupancy, the accuracy for determining nB is noticeably better for a high average depletion (> 80%) than for depletion levels below 10%.

We further demonstrate that measuring binding under ligand-depleting conditions enables the quantification of accessible surface target structures on cells and tissue with both radioligands and fluorescently labelled antibodies. Real-time interaction analysis is not depending on absolute signal values and information is extracted from the generated signal change over time, i.e. the shape of the binding trace^17^. Therefore, knowing the signal contribution per ligand molecule is not necessary when using depletion-based kinetic modelling for extracting the number of binding sites. This is an advantage compared to saturation-based manual methods that require knowledge of the signal contribution per ligand molecule to convert the signal at receptor saturation to a receptor number^18^. For fluorescent ligands, quantitative flow cytometry (QFCM) is typically the method of choice to quantify cell surface antigen expression where calibrated beads coated with a precisely defined number of reference molecules are used to determine the fluorescent signal contribution per antibody molecule, referred to as the MESF (Molecules of Equivalent Soluble Fluorochrome) value^19,20^. Hence, both approaches, radioligand binding saturation assays and QFCM, require that the ratio between ligand concentration and generated signal is calibrated for each experiment. A further challenge with saturation assays is that standard fittings of radioligand binding curves, even when using the depletion model, are not accurate if high ligand depletion (> 50%) is present in the initial concentrations^8^. As neither signal calibration nor saturation is needed when applying depletion-based kinetic modelling for determining surface antigen expression levels, the method described in this paper simplifies the work-flow and minimizes the impact of experimental variables such as the use of different labelling batches and washing steps^20,21^. Moreover, the accessibility of binding sites is quantified in a cellular network instead of on separated cells and thus the presented method can also be applied to tissue samples, which may more accurately reflect the accessible binding sites in vivo*.*

As examples, we assessed the kinetics and quantified the number of receptors per cell experimentally for a collection of 6 tracers, including a low molecular weight compound, peptides and antibodies labelled with radionuclides (Figs. 2, 3) or fluorescent dyes (Fig. 4). We used experimental parameters that drove the system towards ligand depletion, i.e., high cell numbers and low ligand concentrations for the first association phase, while still aiming for a target occupancy above 25% at the end of the last association. In all the investigated systems, the 1:1 model with depletion correction was better at fitting the experimental data than the standard 1:1 model, with the latter displaying deviations that are typical signs of ligand depletion. Depletion-based nB values were in good agreement to the number of binding sites determined through KEX, though it was noted that nB values tended to be higher compared to KEX. These differences might be explained by practical experimental procedures. Cells were harvested after completing LigandTracer experiments to obtain the cell count. Both necessary washing steps and incomplete cell detachment can result in cell loss and thus the actual cell number present during the LigandTracer experiment may be slightly higher than the cell count. However, it should be noted that these differences are small when comparing literature values for receptor expression on cell lines: for example, for HER2 on SKOV3 cells literature values range from 3.3 × 10^5^ molecules/cell^22^ over 1.4 × 10^6^ receptors/cell^23^ and 5.6 × 10^6^ receptors/cell^11^ to 20 × 10^6^ receptors/cell^24^. Our determined expression of 1.9 and 2.1 × 10^6^ receptors/cell with two different antibodies is in the middle of this rather broad range. For PC3-PIP cells, literature stated 4.9 × 10^6^ binding sites/cell, which was assessed by radioligand binding saturation assays^25^. Using the Glu-ureido–based PSMA-Tracer, we calculated it to be 1.2 × 10^7^ binding sites/cells (depletion method) and 8.0 × 10^6^ binding sites/cells (KEX method), and with the PSMA-Ab, the number of available binding sites was quantified to be 3.8 × 10^6^ binding sites/cells (depletion method) and 3.7 × 10^6^ binding sites/cells (KEX method), which is in good agreement with literature. The differences between the numbers obtained with the Glu-ureido–based PSMA-Tracer and the PSMA-Ab can be explained by the antibody binding bivalent and thus occupying to two targets per one ligand molecule. Since the depletion model assumes monovalent binding, this is not automatically taking into account and explains why the antibody binding capacity is roughly half compared to monovalent PSMA-Tracer. In addition, the Glu-ureido–based molecule and PSMA-Ab do not bind to the same binding site and do vary in size, which can affect the number of accessible binding sites in the sample. Furthermore, variations in reported expression levels are in parts due to biological variations and also influenced by the methodology of choice as the number of accessible surface receptors can vary depending on e.g. whether the measurement is performed on separated cells or in situ in a cellular network. Taken together, the variability of the published data and differences in what is actually analysed, points out the need to extend the toolbox for methods that can easily quantify surface marker expression within a relevant biological environment with a minimal number of work-steps and no need for calibration.

Detailed insights on how experimental design affects parameter extraction can be used to improve both assay design as well as the mathematical optimization method used for kinetic fitting. This is a prerequisite for developing more complex kinetic models that can give reliable information about the biological system under investigation. Models that can represent the biological complexity in an adequate manner are of value when relating binding to functional properties, as this knowledge has potential to facilitate rational drug design for improved therapeutics. In this report, we demonstrate that both 1:1 binding kinetics as well as the number of binding sites can be extracted by modelling ligand depletion for real-time binding experiments on cells and tissue. Both quantification of binding and surface marker expression add valuable information during the drug development process and measuring both simultaneously allows to easily relate these two aspects. This is particularly of value for molecules that can change their apparent affinity depending on target expression, such as monoclonal antibodies (mAbs), which are a rapidly growing class of therapeutics^3,26,27^. Importantly, changes in apparent affinity due to avidity effects have been shown to impact the efficiency of their mechanism of action^28–31^ and thus mAbs are a prime example of how number of available binding sites can influence binding kinetics and thus the therapeutic mechanism of action.

## Materials and methods

### Cell culture

All cells were maintained under standard cell culture conditions (37 °C, 5% CO_2_, saturated humidity).

CHO cells stably transfected with human GIPR (gastric inhibitory polypeptide receptor) were obtained from InSCREENeX GmbH (Germany) and cultured in Dulbecco`s Modified Eagle`s Medium/Nutrient Mixture F-12 Ham (Cat.No. D6421, Sigma) supplemented with 5% fetal bovine serum (Cat.No. P30-3306, PAN Biotech), 8 mM L-Glutamine (Cat.No. P04-04,500, PanBiotech), 100 U/ml Penicillin–Streptomycin (Cat.No. P0781, Sigma) and 0.5 mg/ml G418 (Cat.No. 10131035, ThermoFisher).

FAP (fibroblast activation protein α)-transfected HEK-293 cells were purchased from InSCREENeX GmbH (Germany) and maintained in DMEM high glucose medium (Cat.No. P04-04500, Pan Biotech) supplemented with 10% fetal bovine serum (Cat.No. P30-3306, PAN Biotech), 8 mM glutamine (Cat.No.P04-82100, PAN Biotech) and 100 U/ml Penicillin–Streptomycin (Cat.No. P0781, Sigma).

PC3 cells stably transfected with human prostate-specific membrane antigen (huPSMA; PC3-PIP cells) were provided by Clovis Oncology Inc (USA) and cultured in RPMI-1640 containing 8 mM stable glutamine (Cat.No. P04-04500, PanBiotech) supplemented with 10% FBS (Cat.No. P30-3306, PAN Biotech) and 100 U/ml Penicillin–Streptomycin (Cat.No. P0781, Sigma).

SKOV3 cells (HTB-77, ATCC, US) were maintained in RPMI1640 containing stable Glutamine (Cat.No L0498, Biowest) supplemented with 10% FBS (F7524, Sigma-Aldrich) and 100 U/ml Penicillin–Streptomycin (L0022, Biowest).

For LigandTracer Yellow assays, CHO-huGIPR, HEK-huFAP and PC3-PIP cells were detached using Accutase (Cat. No. 423201, Bio Legend) and cell concentrations of detached cells were adjusted to the required concentration (Table 1, SI). Three milliliters of the cell suspension were then dispensed into a poly-D-lysine coated petri dish placed on an inclined support and allowed to attach under standard cell culture conditions until the start of the assay.

For LigandTracer Green assays, SKOV3 cells were detached using 0.25% Trypsin–EDTA (Cat.No 25200, Gibco) and seeded on MultiDishes 2 × 2 (Cat. No.1-04-201-5, Ridgeview Instruments AB, Sweden). Cells were placed in opposite sectors with 9 × 10^5^ cells being seeded in each of the sectors the day prior to experiments, alternatively 7 × 10^5^ cells 2 days prior to the experiment. Before the start of the experiment, the cell media was exchanged to 1.8 mL CO_2_-independent media (Cat. No. 18045-054, Gibco) supplemented with 10% FBS (F7524, Sigma-Aldrich) and 100 U/ml Penicillin–Streptomycin (Cat.No. P0781, Sigma) for each compartment to improve cell adherence during the assay.

### Tissue

PC3-PIP xenograft tissue from mice was obtained from preclinics GmbH (Germany) and stored at − 80 °C. Tissues were allowed to equilibrate for at least 1 h in the cryotome chamber of a Leica 3050 Cryostat before sectioning at − 18 °C (chamber temperature). Sections with 10 µm thickness were attached to a poly-D-lysine coated petri dish and stored at − 80 °C until use for max. 2 weeks. Prior the assay, the tissue was pre-warmed with the petri-dish being inverted inside an incubator (37 °C, 5% CO_2_, saturated humidity) for 5 min. A consecutive slide was cut onto a microscope slide, stained with H&E and imaged with a microscope (Keyence).

### Radiolabeling

The peptide tracers targeting GIPR (GIPR-Tracer), FAP (FAP-Tracer) and the PSMA-targeting urea-based tracer (PSMA-Tracer) were synthesized in-house at 3BP. Qualitative analysis of synthesized batches was performed using LC-TOF. Purity of batches used was at least 90%. For radiolabeling, 200 μM compound stock solutions were prepared by dissolution in 0.1 M HEPES, aliquoted and stored at − 20 °C.

At the start of the radiolabeling volumes of radionuclide solution (^111^InCl_3_ in 20 mM HCl) containing required amounts of activity were transferred into 1.5 mL LoBind Eppendorf vials. Then, 1 M ammonium acetate buffer pH 5.5 was added to yield a final buffer concentration of 0.1 M followed by 1.0 nmol of the 3BP compound per 5 or 30 MBq radionuclide. Subsequently, the mixture was heated to 90 °C for 15 min, followed by cooling for 5 min. Finally, 15 µL of 200 mg/mL ascorbic acid solution, 2.5 µL of 5 mg/mL DTPA and 2.5 µL of 5% TWEEN-20 per 100 µL reaction mixture were added, resulting in the concentrated study solution.

For quality control, an aliquot of the labeling solution was diluted 1:40 with 0.1% TWEEN-20 in 0.1 M ammonium acetate buffer pH 5.5. Five microliters of the diluted labeling solution were injected onto a Poroshell SB-C18 2.1 × 50 mm, 2.7 μm column. HPLC analysis was performed as follows: Eluent A: H_2_O, 0.1% TFA, Eluent B: MeCN, gradient from 5% B to 70% B within 15 min, flow rate of 0.5 mL/min; detector: NaI, DAD 210 nm. The peak eluting with the dead volume represents free radionuclide, the peak eluting with the peptide-specific retention time as determined with a non-labeled sample represents radiolabeled compound. The retention times of the ^111^In-labeled products according to HPLC are stated in Suppl Table 2. The radiochemical purity of the study formulations was measured to be on average 94% for ^111^In-GIPR-Tracer, 81% for ^111^In-FAP-Tracer and 97% for ^111^In-PSMA-Tracer at the end of synthesis for all assays.

The recombinant human monoclonal anti-PSMA antibody mAb HuJ591-GS (Biorbyt, Cat. No. orb1141266) was linked to DTPA via maleimide–thiol conjugation using Maleimide-DTPA (synthesized in-house at 3BP) and Tris(2-carboxyethyl)phosphin–hydrochlorid (TCEP, Sigma, Cat. No. C4706-10G). The antibody conjugate was then dissolved in ammonium acetate buffer pH 5.5 to obtain a stock solution of 1 mg/mL, which was aliquoted and stored at − 20 °C. Before use, the aliquots were thawed and centrifuged for 60 s at 13.400 rpm. For the radiolabeling, volumes of radionuclide solution (^111^InCl_3_ in 20 mM HCl) containing required amounts of activity were transferred into 1.5 mL LoBind Eppendorf vials. Then, 1 M ammonium acetate buffer pH 5.5 was added to yield a final buffer concentration of 0.1 M followed by 50 µg of the DTPA labeled antibody per 10 MBq radionuclide. Subsequently, the mixture was heated to 40 °C for 60 min with 400 rpm. For quality control, an aliquot of the labeling solution was diluted 1:40 in 1 × PBS and 1–2 µL of this dilution was applied on iTLC-SG strips (Agilent, SGI0001, ca. 7.6 × 2.3 cm), which were pre-activated for ~ 30 min in drying oven at 110 °C. The strips were then developed in citrate-dextrose solution (Sigma, C3821) for at least 15 min, allowed to dry slightly and then cut into three pieces. The radioactivity associated with each piece was measured with a gamma-counter (Perkin Elmer, USA). The results were analyzed as follows:

- Rf = 0: DTPA labeled antibody
- Rf = 1: free radionuclide and colloids
- Incorporation yield [%] = 100 * (Counts on lower part)/total counts

The radiochemical purity of the study formulations was measured to be in average 98%.

### Fluorescent labeling

Therapeutic antibodies Trastuzumab and Pertuzumab (Apoteket AB, Sweden) were labeled with ATTO-488 NHS-Ester (Cat.No AD488-31. AttoTech, Germany) according to the manufacturer’s instructions. Labeled antibodies were purified into PBS using NAP-5 columns (Cytivia, Sweden) and their concentration and degree of labeling (DOL) quantified using a NanoPhotometer (Implen) with cuvette. For Trastuzumab, labeling with Atto488 resulted in an antibody concentration of 216 µg/mL with a DOL of 1.78 and for Pertuzumab the resulting antibody concentration was 200 µg/mL with DOL of 1.70.

### LigandTracer assay

Real-time ligand-receptor interaction measurements on live cells were performed using LigandTracer Yellow (Ridgeview Instruments, Uppsala, Sweden) for radioactive compounds^32^ and LigandTracer Green (Ridgeview Instruments) for fluorescent compounds^33^. Briefly, the instrument comprises an inclined, rotating support, on which a standard petri dish can be placed, and a detector mounted above the upper part of the support area. For LigandTracer Yellow the detection unit is scintillator-based whereas LigandTracer Green has an optical detection unit. In this study the Blue-Green detection unit with an excitation wavelength of 488 nm and emission wavelength of 535 nm was used. Ligand-target interactions were assessed by placing a petri dish with cells or tissue in a defined area onto the inclined support and adding medium containing the labelled ligand. During the measurement, the dish is rotating and the radioactive or fluorescent signal is detected from two or more areas of the dish: The areas containing the cells of interest are denoted the target areas and the area devoid of cells (or containing cells negative for the target of interest) functions as the reference area. Through the rotation combined with the inclination, the liquid containing unbound ligand is outside of the detection zone and repeated measurements of both the target and the reference area allow to determine the level of radioactivity or fluorescence accumulated or retained by the cells in real-time with high temporal resolution. For measurements with LigandTracer Yellow a standard cell culture petri-dish was used and LT binding analysis was performed at room temperature in the one target, one background, fast interaction mode with the detection time being set to 15 s and the detection delay time to 5 s. The assay volume was kept constant at 3 mL during the entire length of the experiments. The signal from the reference area (background) was automatically subtracted from the target area in LigandTracer control.

For measurements with LigandTracer Green MultiDish 2 × 2, which allows to perform 2 independent assays in parallel, was used. Data were recorded from 2 target areas and 2 reference areas during each rotation with a detection time of 15 s and detection delay time of 3 s. The assay volume was kept constant at 1.8 mL per compartment during the entire experiment duration. The signal from the reference area (background) was automatically subtracted from the corresponding target area in LigandTracer control.

Independent of instrument type, first a baseline was established with the appropriate cell culture media as mentioned above. Then labelled ligand was added at three consecutive concentrations (ligand concentrations used for the different interactions are specified in each graph). The association solutions were replaced for each association phase in order to know the starting ligand concentration for each association phase independent of ligand depletion. At the start of the dissociation phase the incubation solution was replaced with cell culture media not containing any free ligand.

After LigandTracer experiments cells were detached from the plates using either trypsin–EDTA (SKOV3 cells) or accutase (CHO-huGIPR, HEK-huFAP and PC3-PIP cells) and counted (automated cell counter TC20, Bio-Rad Laboratories, Germany). The same cell suspension was used for estimating the number of receptors via kinetic extrapolation method (KEX).

### Estimating the number of receptors per cell using the kinetic extrapolation method (KEX)

For estimating the number of receptors per cell using the KEX method, cells were detached from the plates after the LigandTracer experiment using accutase. The obtained cell suspension was used to determine the number of cells as well as the radioactivity using a gamma counter (Perkin Elmer, USA) and the KEX method was applied as previously described^11^.

### Real-time interaction analysis

The binding kinetics of an interaction between a ligand, L, and a target, T, can be modelled according to the 1:1 model (Eq. (1)), where [T] denotes the concentration of free target, [LT] denotes the concentration of the ligand-target complex and [L] denotes the free ligand concentration. While the concentration of ligand-target complexes and free targets change over time as the two molecules interact, the ligand concentration is assumed to be constant for each association phase Eq. (1). The association rate constant k_a_, also referred to as the on-rate describes the process of ligand-target complex formation and has the units s^−1^ M^−1^. The dissociation rate constant k_d_, also referred to as the off-rate, describes the stability of the interaction and has the units s^−1^. The affinity K_D_ is calculated through the ratio of the rate constants k_d_/k_a_.

1 $$\frac{d[LT]}{dt}={k}_{a}\left[\mathrm{L}\right]\left[\mathrm{T}\right]-{k}_{d}\left[LT\right],$$

$$\frac{d[T]}{dt}={-k}_{a}\left[\mathrm{L}\right]\left[\mathrm{T}\right]+{k}_{d}\left[LT\right].$$

The 1:1 model approximates the ligand concentration to be constant during an association phase, when this approximation is not valid due to excess ligand binding, for example when the binders are strong and/or the target concentration is high, the 1:1 model can be extended to account for the decrease in free ligand concentration during the course of the experiment (Eq. (2)). The depletion corrected 1:1 model is also simply referred to as the depletion model.

2 $$\frac{d\left[L\right]}{dt}={-k}_{a}\left[L\right]\left[T\right]+{k}_{d}\left[LT\right],$$

$$\frac{d\left[LT\right]}{dt}={k}_{a}\left[L\right]\left[\mathrm{T}\right]-{k}_{d}\left[LT\right],$$

$$\frac{d[T]}{dt}={-k}_{a}\left[\mathrm{L}\right]\left[T\right]+{k}_{d}\left[LT\right].$$

The concentration of free target can be expressed relative to the total target concentration [T_tot_] minus the concentration of ligand-target complex [LT], which eliminates the need to express the change in free target concentration as a separate differential  (Eq. (3)).

3 $$\frac{d\left[L\right]}{dt}={-k}_{a}\left[L\right]\left(\left[{T}_{tot}\right]-\left[LT\right]\right)+{k}_{d}\left[LT\right],$$

$$\frac{d\left[LT\right]}{dt}={k}_{a}\left[L\right]\left(\left[{T}_{tot}\right]-\left[LT\right]\right)-{k}_{d}\left[LT\right].$$

For real-time binding measurements, as e.g. SPR and LigandTracer assays, the recorded signal is proportional to the concentration of ligand-target complex, and therefore [LT] can be expressed as the measured signal B and [T_tot_] as B_max_ which denotes the theoretical maximum signal, corresponding to the signal at target saturation (Eq. (4)) Thereby the equations are no longer expressed in concentrations (Eq. (3)) but in recorded signal units (Eq. (4)) and thus the concentration-signal proportionality constant α needs to be introduced.

4 $$\alpha \frac{d\left[L\right]}{dt}={-k}_{a}\left[L\right]\left({B}_{max}-B\right)+{k}_{d}B$$

$$\frac{dB}{dt}={k}_{a}[L]\left({B}_{max}-B\right)-{k}_{d}B$$

We can express the ligand concentration [L] in number of ligand molecules L, which introduces the assay volume (vol) and Avogadro’s constant N_A_ (Eq. (5)). As we convert the ligand concentration to number of molecules, signal units need to be converted to number of molecules instead of concentration units. This is done by replacing α with the ratio between the maximum signal B_max_ and the number of available ligand binding sites nB (which is assumed to be equal to the number of targets for a 1:1 interaction) (Eq. (6)).

5 $$\left[\mathrm{L}\right]= \frac{\mathrm{L}}{{\mathrm{N}}_{\mathrm{A}}*\mathrm{vol}}$$

6 $$\alpha =\frac{{B}_{max}}{nB}$$

Hence, transforming the ligand concentration [L] to number of ligand molecules L and multiplying Eq. (4) by nB/B_max_ as described by Eq. (6) leads to Eq. (7):

7 $$\frac{dL}{dt}=-\left({\mathrm{k}}_{\mathrm{a}}\frac{\mathrm{L}}{{\mathrm{N}}_{\mathrm{A}}*\mathrm{vol}}\left({B}_{max}-B\right)-{k}_{d}\mathrm{B}\right) *\frac{nB}{{B}_{max}}$$

$$\frac{\mathrm{dB}}{\mathrm{dt}}={\mathrm{k}}_{\mathrm{a}}\frac{\mathrm{L}}{{\mathrm{N}}_{\mathrm{A}}*\mathrm{vol}}\left({B}_{max}-B\right)-{k}_{d}\mathrm{B}$$

The differential describing the change in ligand molecules over time can be rewritten more compactly (Eq. (8)):

8 $$\frac{dL}{dt}=-\frac{\mathrm{dB}}{\mathrm{dt}} *\frac{nB}{{B}_{max}}$$

$$\frac{\mathrm{dB}}{\mathrm{dt}}={\mathrm{k}}_{\mathrm{a}}\frac{\mathrm{L}}{{\mathrm{N}}_{\mathrm{A}}*\mathrm{vol}}\left({B}_{max}-B\right)-{k}_{d}\mathrm{B}$$

The initial value of L, ${L}_{0,i}$, for each concentration interval $con{c}_{i}$, is the added ligand concentration at the start of each concentration interval (Eq. (9)):

9 $${L}_{0,i}=con{c}_{i}*vol*{N}_{A}$$

Ligand depletion is visible in the shape of the binding curve as a reduction in free ligand concentration results in the formation of less ligand-target complexes and shorter time to equilibrium (and thus more curvature) than what would be expected according to a 1:1 model assuming a constant free ligand concentration. In the model formulation as in Eq. (8) this information is fitted as the nB parameter, the total number of binding sites or targets. By performing a cell count, the number of receptors per cell can be calculated.

All experimental binding traces recorded with LigandTracer were fitted in TraceDrawer 1.9.2 using the 1:1 model and the 1:1 depletion model. Default starting guesses and scopes were used, except for the following parameters of the depletion model: nB was set to global with a start value of 1 × 10^10^ and the volume was set constant to the corresponding assay volume used, i.e. 3 mL for experiments performed with radiolabeled compounds and 1.8 mL for experiments performed with fluorescent compounds.

### Simulations

Kinetic binding curves were simulated using an implementation of the 1:1 depletion model (Eq. (8)) in C +  + and Visual Basic for solving the differential equation, which is the same mathematical approach as implemented in the commercially available software TraceDrawer 1.9.2 that was used for evaluating the experimental data. For on-rates, off-rates and number of receptors random parameters were drawn from a uniform distribution in log-scale with the limits given in Suppl Table 3. The parameter B_max_ was set constant to 100 for all curves and the assay volume was kept constant to 3 mL. Five concentration series with three association phases each were used in the simulations, according to Suppl Table 4. Additionally, three concentration series with two association phases each were simulated according to Suppl Table 5.

As described previously, decent curvature is needed for successful determination of kinetic parameters from real-time binding traces^16^. Therefore, simulated curves that resulted in an average curvature of less than 20% or a maximum curvature of more than 95% were discarded (see definitions below). The assumption was made, that for LigandTracer experiments, at least 10% of target saturation is reached in the last association phase. Therefore, simulated curves where the maximum signal did not reach 10% of B_max_ were excluded from further analysis. Furthermore, simulated curves with less than 30% difference in signal at the end of the last association phase and at the end of the first association phase were also excluded to increase the likelihood that each association phase adds additional signal and thus additional information. Filtering the simulated data in this way resulted in 244 simulated curves with three association phases and 148 simulated curves with two association phases that were deemed having suitable information content for fitting kinetic models. The same math library as is implemented in TraceDrawer 1.9.2 (Ridgeview Instruments AB) was used to fit the simulated curves using the 1:1 model and the 1:1 depletion model. The default TraceDrawer starting guesses and scopes were used; except for the following parameters in the 1:1 depletion model: nB was set to global with starting value 1 × 10^10^ and the volume was set constant to 3 mL.

Outliers, with regards to the relative parameter error (see definition below), were removed using Grubbs method and a threshold of 0.01% prior to further processing and visualization. Data processing was performed with Matlab 2022b and visualization was performed with Prism 8.0.1.

### Definition of measures

Several different measures were calculated from the simulated and fitted parameters.

#### Curvature

**Figure 5:**
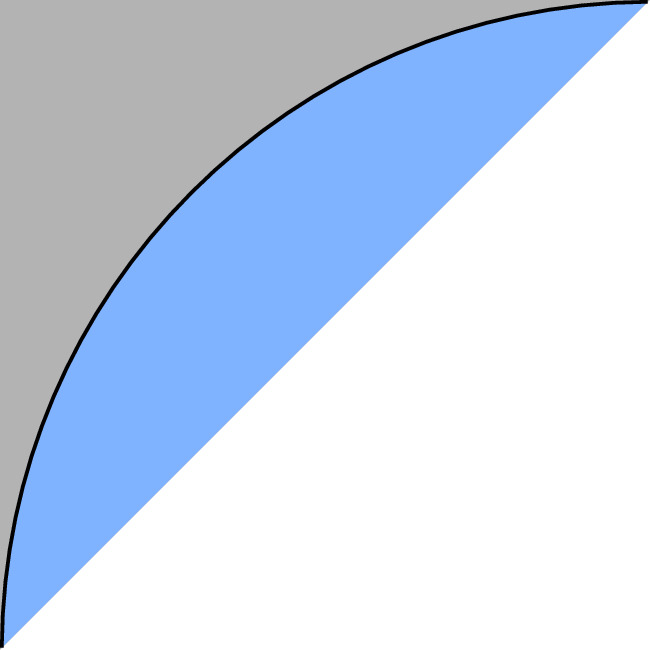
The % of the blue area represents the curvature for the association phase.

The curvature measure for a binding trace was defined for each association phase as the % of the area of a triangle with corners at (${t}_{0}$, ${B}_{{t}_{0}}$), (${t}_{max}$, ${B}_{{t}_{max}}$) and (${t}_{0}$, ${B}_{{t}_{max}}$) covered by the trace as explained by Fig. 5. The average curvature of all association phases was defined as the average curvature per simulated binding trace, the maximum curvature as the association phase with most curvature and the minimum curvature as the association phase with least curvature.

#### Depletion

The degree of ligand depletion was calculated for each association phase and defined as the percentage of unbound ligand that gets depleted from the solution until the end of that association phase.

10 $$Degree \,of\, depletion\, (\%)\, =\,\frac{{\left[L\right]}_{depleted}}{{\left[L\right]}_{added}} * 100.$$

The change in ligand concentration through depletion was calculated using Eq. (11) where ${Y}_{start}$ is the signal at the start of the current association phase and ${Y}_{end}$ is the signal at the end of the current association phase.

11 $${[L]}_{depleted}=\left(\frac{{Y}_{end}}{Bmax}- \frac{{Y}_{start}}{Bmax}\right)* \frac{nB}{{N}_{A}*vol}.$$

The mean depletion D_mean_ for a binding trace was calculated by averaging the degree of ligand depletion for all association phases.

#### Relative parameter error

The parameter errors were calculated as follows:

12 $$parameter\, error\,=\,\frac{Abs\left(paramete{r}_{simulated}-paramete{r}_{fitted}\right)}{paramete{r}_{simulated}} * 100$$

## Supplementary Information

Supplementary Tables.

## Data Availability

All datasets included in the study are available from the corresponding author upon request.
