# Supplementary material for: Modelling ligand depletion for simultaneous affinity and binding site quantification on cells and tissue
Source: Sci Rep. 2023 Jun 20;13:10031. doi: 10.1038/s41598-023-37015-1 (PMC10282064; doi:10.1038/s41598-023-37015-1)
Supplement: Supplementary file 1 — Supplementary Tables. [file 41598_2023_37015_MOESM1_ESM.pdf]

Supplementary Information

Modelling ligand depletion for simultaneous affinity and binding site quantification on cells and tissue

Judith Weber[1], Klara Djurberg[2], Sara Lundsten Salomonsson[2,3], Maria Kamprath[1], Aileen Hoehne[1], Hadis Westin[2], Fernanda Vergara[1], Sina Bondza*[2,3]

[1] 3B Pharmaceuticals GmbH, Berlin, Germany
[2] Ridgeview Instruments AB, Uppsala, Sweden
[3] Department of Immunology, Genetics and Pathology, Uppsala University, Uppsala, Sweden
*Corresponding author: s.bondza@ridgeviewinstruments.com

Supplementary Tables

Table 1. Cell concentration [cells/mL] for plates used for experiments with varying cell numbers between experiments. Cells were seeded one day prior the experiment. 3 mL cell suspension was used for seeding per plate.

	CELL CONCENTRATION [CELLS/ML]							
**CELL LINE**	**Exp.1**	**Exp.2**	**Exp.3**	**Exp.4**	**Exp.5**	**Exp.6**	**Exp.7**	**Exp.8**
HEK-FAP	1E+06	5E+05	1E+06	5E+05	1E+06	2E+06	1E+06	2E+06
PC3-PIP	1E+06	5E+04	1E+06	5E+05	1E+05	5E+05	n.a.	n.a.

Table 2. HPLC retention times of radiolabeled test compounds

TEST COMPOUND	RETENTION TIME [MIN]
^{111}In-GIPR-TRACER	6.9
^{111}In-FAP-TRACER	7.6
^{111}In-PSMA-TRACER	5.8

Table 3. Parameter intervals for simulation of curves

PARAMETER	LOWER LIMIT	UPPER LIMIT
Log(k_a)	3	6
Log(k_d)	-6	-3
Log(nB)	10	14

Table 4. Concentration series of simulated three association phases data

**CONCENTRATION ASSOCIATION 1 (nM)**	**CONCENTRATION ASSOCIATION 2 (nM)**	**CONCENTRATION ASSOCIATION 3 (nM)**
0.3	1	3
1	3	5
1	10	20
1	3	9
0.3	1	10

Table 5. Concentration series of simulated two association phases data

**CONCENTRATION ASSOCIATION 1 (nM)**	**CONCENTRATION ASSOCIATION 2 (nM)**
1	3
1	9
3	9
